# Overall survival of neoadjuvant versus adjuvant systemic treatment for breast cancer in Brazil

**DOI:** 10.11606/s1518-8787.2025059006671

**Published:** 2025-12-01

**Authors:** Carolina Zampirolli Dias, Carolina Campos Vieira de Sousa, Thais Piazza, Ilka Afonso Reis, Augusto Afonso Guerra, Mariangela Leal Cherchiglia

**Affiliations:** I Universidade Federal de Minas Gerais. Faculdade de Medicina. Programa de Pós-Graduação em Saúde Pública. Belo Horizonte, MG, Brasil; II Universidade Federal de Minas Gerais. Faculdade de Medicina. Belo Horizonte, MG, Brasil; III Ministério da Saúde. Departamento de Gestão e Incorporações de Tecnologias em Saúde. Brasília, DF, Brasil; IV Universidade Federal de Minas Gerais. Instituto de Ciências Exatas. Departamento de Estatística. Belo Horizonte, MG, Brasil; V Universidade Federal de Minas Gerais. Faculdade de Farmácia. Departamento de Farmácia Social. Belo Horizonte, MG, Brasil; VI Universidade Federal de Minas Gerais. Faculdade de Medicina. Departamento de Medicina Preventiva e Social. Belo Horizonte, MG, Brasil

**Keywords:** Breast Neoplasms, Surgical Oncology, Neoadjuvant Therapy, Survival Rate

## Abstract

**OBJECTIVE:**

Compare the overall survival of women with non-metastatic breast cancer who received neoadjuvant systemic therapy followed by surgery *versus* those who underwent surgery followed by adjuvant systemic therapy.

**METHODS:**

A nationwide retrospective cohort study was conducted using real-world data from 2008 to 2015 available on the Brazilian Unified Health System (SUS). Women aged ≥ 18 years with breast cancer undergoing surgery or neoadjuvant systemic therapy as their first treatment between 2008 and 2010 were included. Cohorts were matched using propensity score matching in a 1:1 ratio and assessed 5-year overall survival using Kaplan-Meier, compared by log-rank test and hazard ratios (HR) using Cox proportional HR model.

**RESULTS:**

A total of 23,331 women began treatment for breast cancer in SUS with neoadjuvant systemic therapy (n = 6,040) and surgery (n = 17,291). In the matched cohorts (n = 6,040 in both groups), more deaths occurred among those who received neoadjuvant systemic therapy compared with surgery as first treatment (37.3% and 19.6%, respectively; p < 0.001). Overall survival after five years was 0.641 in neoadjuvant systemic therapy and 0.816 in the surgery group (p < 0.001). For both groups, older patients (≥ 70 years) living in northern and midwestern Brazil, in municipalities with low and medium HDI, and self-declared as Black presented the lowest overall survival probabilities. Use of hormone therapy after surgery and conservative surgery instead of mastectomy were associated with higher survival. HR was 5.13 (95%CI: 2.95–8.88) in stage I, 1.57 (95%CI: 1.27–1.95) in stage II, and 1.38 (95%CI: 1.26–1.50) in stage III.

**CONCLUSION:**

Women who underwent surgery as first treatment had a significantly higher 5-year overall survival compared with those who received neoadjuvant systemic therapy. Socioeconomic and demographic factors influenced survival outcomes.

## INTRODUCTION

Breast cancer is the most common neoplasm affecting women and has the highest mortality rate worldwide^1^. Considered a multifactorial disease, it is influenced by environmental, behavioral, genetic, and endocrine factors^2^. Population aging is a key factor in the increasing incidence and mortality from breast cancer worldwide^3^.

In Brazil, breast cancer is the most prevalent type of cancer among women across all regions^4^. Between 2023 and 2025, 74,000 new cases are expected, representing 66.54 cases per 100,000 women^4^. Given this context, early diagnosis coupled with increasingly effective treatments is crucial for enhancing patient quality of life and for reducing the morbidity and mortality associated with the disease^5,6^.

Guidelines recommend surgery as the primary treatment and control of non-metastatic breast cancer^7,8^. The tumor can be excised with surrounding breast tissue, preserving the breast, or the breast can be completely removed (mastectomy)^7^. The choice of the type of the surgical procedure considers clinical criteria and factors related to the size and extent of the tumor^9^. However, a wide range of surgical procedures of varying complexity and infrastructure are required^8^. If indicated, radiotherapy and adjuvant systemic treatment (AST) may be administered after surgery^7,9^. Before surgery, neoadjuvant systemic therapy (NST) can also be administered^9,10^.

NST has emerged as a viable treatment option for patients with locally advanced breast cancer or those for whom surgical resection is not an immediate possibility. It effectively reduces tumor size and decreases nodal involvement. Additionally, literature suggests that NST can serve as an alternative therapy for patients with lymph node-negative breast cancer (cN0) and unfavorable prognostic factors, particularly when systemic therapy is deemed necessary before surgery^11^. Moreover, it helps identify patients with chemotherapy-resistant disease, providing early information on the potential effectiveness of the systemic treatment used and reducing the risk of disease recurrence^10^.

Fortunately, breast cancer treatment has advanced over the years, achieving even better outcomes than those seen before^9^. Studies comparing women with non-metastatic breast cancer who underwent systemic treatment before (neoadjuvant) or after (adjuvant) surgery found no statistically significant difference in overall survival (OS) and in the death rate from breast cancer or other causes^13^.

Brazil’s territorial extension and diversity impact the type and quality of health services available and, consequently, the outcomes^16^. Thus, analyzing nationwide real-world data such as those from Brazilian Unified Health System (SUS) can help to better understand the results achieved in terms of survival according to treatment received, helping to formulate public policies that guarantee equitable access to effective treatments.

Our study compares the OS of women with non-metastatic breast cancer who received NST followed by surgery *versus* those who underwent surgery followed by AST in SUS.

## METHODS

### Study Design and Data Source

We conducted a nationwide retrospective cohort study using data available on the National Health Database centered on the individual, constructed via probabilistic-deterministic linkage of SUS administrative databases^17^. This database integrates data from the main SUS Information Systems, including, but not limited to, the Outpatient Information System (SIA), Hospital Information System (SIH), and the Mortality Information System (SIM)^17^. SIA and SIH receive and store information about the population’s use and payment of health services carried out based on procedures performed at the outpatient and hospital level. SIM gathers data on deaths in the country, fed with information from the death certificate^18^. The database was anonymized, and a unique identification code identifies each individual^17^.

### Eligibility Criteria

Inclusion criteria were defined as women aged ≥18 years old, diagnosed with breast cancer (ICD-10: C50.0, C50.1, C50.2, C50.3, C50.4, C50.5, C50.6, C50.8, C50.9), treated at a SUS outpatient setting, who underwent surgery (mastectomy or conservative surgery), in which surgery or NST was the first treatment administered, between January 2008 and November 2010. Patients diagnosed at stages 0, IV, or not reported, who underwent radiotherapy on the same date as the initial treatment with NST or surgery were excluded. Additionally, women who underwent surgery as their initial treatment and did not receive adjuvant systemic therapy afterward, or received another systemic therapy after NST and before surgery, were also excluded. Patients without race/skin color information, including Asian or Indigenous individuals were not included due to potential under-representation19.

### Outcomes and Variables of Interest

Our primary outcome was to compare the 5-year OS rate of women with breast cancer treated with NST followed by surgery with those who underwent surgery followed by AST at SUS. Additionally, we examined the frequency of conservative surgery and mastectomy in both groups for exploratory purposes.

Sociodemographic and clinical characteristics were also described, including age at treatment initiation (18–39, 40–49, 50–59, 50–69 and ≥ 70 years), self-reported race or skin color (White, Black or Brown/Mixed-race) and region of residence (North, Northeast, Central-West, South, and Southeast). We assessed the municipality of residence according to their rural or urban classification defined according to the demographic density, the location in relation to the main urban centers and population size (rural, intermediate or urban); Human Development Index (HDI) (very high, high, medium, low and very low); and per capita income. Presence of comorbidities was assessed using the Elixhauser score in the first year of treatment (yes/no), stage at diagnosis according to TNM classification (I, II or III), tumor localization by the International Statistical Classification of Diseases and Related Health Problems 10th Revision (ICD-10) (unspecified site: C50, C50.9; nipple and areola: C50.0; central portion of breast: C50.1; inner/outer quadrant: C50.2, C50.3, C50.4, C50.5; axillary portion of breast: C50.6; with invasive lesion: C50.8), if the patients were treated with hormone therapy, chemotherapy or anti-HER2 drugs after surgery (yes/no), and if they received radiotherapy (yes/no).

### Statistical Analysis

Descriptive analyses used frequency distributions for categorical variables and median and interquartile range (IQR) for continuous variables. Groups were compared using Pearson’s chi-square test for categorical variables and Mann-Whitney test for continuous variables.

To compare the groups (NST followed by surgery versus surgery followed by AST) controlling potential confounders, we matched women from each group using propensity score matching (PSM). PSM calculation included the covariates considered potential cofounders: age and year of treatment initiation, region of residence, race/skin color, and use of hormone therapy. Importantly, receiving hormone therapy suggests that the patient had a hormone receptor-positive tumor. Not receiving it indicates a hormone receptor-negative tumor or a patient with positive hormone receptor tumor who did not receive adequate treatment. We used the nearest neighbor method, in a 1:1 proportion, on the R package “MatchIt.” Frequency distributions of the paired variables were verified by Pearson’s chi-square test.

The 5-year OS was estimated as the time elapsed, in months, between the date of treatment onset and event occurrence (any cause death) or end of follow-up (December 1, 2015). We considered the earliest date available in the SIH and SIA databases for the first treatment procedure (surgery or NST). Survival curves were estimated using Kaplan-Meier and log-rank test compared the curves, adopting a significance level of 5%. Hazard ratios (HR) and their confidence intervals were assessed by Cox proportional HR model. Comorbidities in the first year of treatment assessed by Elixhauser score and stage at diagnosis were considered cofounders and used as adjustment variables in the regression models. Model adequacy was assessed by Schoenfeld residuals analysis. All analyses were performed using R software, on RStudio graphical interface.

### Ethics

The ethics committee of the Universidade Federal de Minas Gerais (CAAE 16334413.9.0000.5149) approved the study. All data used in the study were anonymized, and the provision of a consent form was not required.

## RESULTS

**Figure 1:**
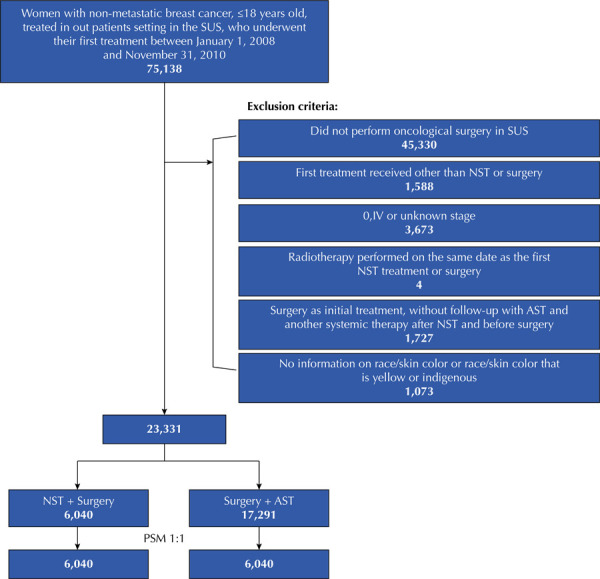
Flowchart of eligibility criteria.

SUS: Brazilian Unified Health System; NST: Neoadjuvant Systemic Therapy; AST: Adjuvant Systemic Treatment; PSM: Propensity Score Matching.

Between 2008 and 2010, 23,331 women started treatment for breast cancer in the SUS undergoing NST followed by surgery (n = 6,040) and surgery followed by AST (n = 17,291). After pairing the cohorts, each group included 6,040 women (Figure 1).

PSM distance achieved a standard mean difference of 0.0062 and the variables paired p > 0.05, indicating that groups were not statistically different (Table 1). Median age of all participants was 54.0 years (IQR = 46.0–64.0), being higher for those who underwent surgery first (56.0, IQR = 47.0–66.0) and lower for those who received NST (51.0, IQR = 43.0–59.0). After matching the cohorts, the median age was 51 years in both groups. Participants were predominantly white (58.0% in NST followed by surgery *versus* 59.8% in surgery followed by AST), living in southeastern Brazil (47.3% and 48.2%, respectively), in urban (82.6% and 83.7%, respectively) and high HDI (60.6% and 58.7%, respectively) municipalities (Table 1).

**Table 1:** Characteristics of patients included in the study, Brazil, 2008–2014.

	Total n = 23,331	Before PSM			After PSM	
		NST + surgery n = 6,040	Surgery + AST n = 17,291	p-value	Surgery + AST n = 6,040	p-value
Age at treatment initiation (years), median (IQR)	54.0 (46.0–64.0)	51.0 (43.0–59.0)	56.0 (47.0–66.0)	< 0.001	50.0 (43.0–59.0)	0.390
Age range, n (%)				< 0.001		0.757
18 to 39	2,345 (10.1)	897 (14.9)	1,448 (8.37)		909 (15.0)	
40 to 49	6,084 (26.1)	1,916 (31.7)	4,168 (24.1)		1,960 (32.5)	
50 to 59	6,475 (27.8)	1,743 (28.9)	4,732 (27.4)		1,736 (28.7)	
60 to 69	4,945 (21.2)	1,067 (17.7)	3,878 (22.4)		1,048 (17.4)	
≥70	3,482 (14.9)	417 (6.90)	3,065 (17.7)		387 (6.41)	
Race/skin color, n (%)				< 0.001		0.089
White	14,441 (61.9)	3,506 (58.0)	10,935 (63.2)		3,560 (58.9)	
Black	1,454 (6.23)	452 (7.48)	1,002 (5.79)		391 (6.47)	
Brown (Mixed-race)	7,436 (31.9)	2,082 (34.5)	5,354 (31.0)		2,089 (34.6)	
Region of residence, n (%)				< 0.001		0.172
North	770 (3.30)	212 (3.51)	558 (3.23)		178 (2.95)	
Northeast	4,554 (19.5)	1,335 (22.1)	3,219 (18.6)		1,356 (22.5)	
Southeast	11,947 (51.2)	2,859 (47.3)	9,088 (52.6)		2,909 (48.2)	
South	4,777 (20.5)	1,155 (19.1)	3,622 (20.9)		1,168 (19.3)	
Midwest	1,283 (5.50)	479 (7.93)	804 (4.65)		429 (7.10)	
Municipality classification, n (%)				< 0.001		0.280
Intermediate	1,214 (5.20)	362 (5.99)	852 (4.93)		344 (5.70)	
Rural	2,424 (10.4)	687 (11.4)	1,737 (10.0)		640 (10.6)	
Urban	19,693 (84.4)	4,991 (82.6)	14,702 (85.0)		5,056 (83.7)	
Human Development Index, n (%)				< 0.001		0.037
Low and very low (0.000 to 0.599)	886 (3.80)	256 (4.24)	630 (3.64)		275 (4.55)	
Medium (0.600 to 0.699)	3,434 (14.7)	996 (16.5)	2,438 (14.1)		980 (16.2)	
High (0.700 to 0.799)	14,232 (61.0)	3,662 (60.6)	10,570 (61.1)		3,543 (58.7)	
Very high (≥ 0.800)	4,779 (20.5)	1,126 (18.6)	3,653 (21.1)		1,242 (20.6)	
Municipality *per capita* income, median (IQR)	1,085 (804–1,599)	1,040 (791–1,494)	1,102 (811–1,639)	< 0.001	1,067 (791–1,599)	0.007
Comorbidities in the first year of treatment^a^, n (%)				< 0.001		0.002
No	22,664 (97.1)	5,932 (98.2)	16,732 (96.8)		5,881 (97.4)	
Yes	667 (2.86)	108 (1.79)	559 (3.23)		159 (2.63)	
Local of the tumor^b^, n (%)				< 0.001		< 0.001
Unspecified location/other	6,609 (28.3)	2,844 (47.1)	3,765 (21.8)		1,170 (19.4)	
Nipple and areola	2,727 (11.7)	778 (12.9)	1,949 (11.3)		773 (12.8)	
Central portion of the breast	2,551 (10.9)	455 (7.53)	2,096 (12.1)		717 (11.9)	
Inner/outer quadrant of the breast	4,106 (17.6)	1,470 (24.3)	2,636 (15.2)		985 (16.3)	
Axillary portion of the breast	2,006 (8.60)	146 (2.42)	1,860 (10.8)		722 (12.0)	
With invasive lesion	5,332 (22.9)	347 (5.75)	4,985 (28.8)		1,673 (27.7)	
Stage at diagnosis, n (%)				< 0.001		< 0.001
I	3,488 (15.0)	45 (0.75)	3,443 (19.9)		1,053 (17.4)	
II	8,611 (36.9)	454 (7.52)	8,157 (47.2)		2,741 (45.4)	
III	11,232 (48.1)	5,541 (91.7)	5,691 (32.9)		2,246 (37.2)	
Received radiotherapy, n (%)				< 0.001		0.008
Yes	15,578 (66.8)	4,828 (79.9)	10,750 (62.2)		4,943 (81.8)	
No	7,753 (33.2)	1,212 (20.1)	6,541 (37.8)		1,097 (18.2)	
Received hormone therapy after surgery, n (%)				< 0.001		0.826
Yes	16,731 (71.7)	3,313 (54.9)	13,418 (77.6)		3,326 (55.1)	
No	6,600 (28.3)	2,727 (45.1)	3,873 (22.4)		2,714 (44.9)	
Received anti-HER2 drugs after surgery, n (%)				0.836		0.174
Yes	61 (0.26)	17 (0.28)	44 (0.25)		27 (0.45)	
No	23,270 (99.7)	6,023 (99.7)	17,247 (99.7)		6,013 (99.6)	
Year of treatment initiation, n (%)				< 0.001		0.537
2008	8,305 (35.6)	2,015 (33.4)	6,290 (36.4)		2,072 (34.3)	
2009	7,982 (34.2)	2,047 (33.9)	5,935 (34.3)		2,009 (33.3)	
2010	7,044 (30.2)	1,978 (32.7)	5,066 (29.3)		1,959 (32.4)	
Follow-up, median (IQR)	72.0 (61.0–83.0)	66.0 (31.0–79.0)	73.0 (63.0–84.0)	< 0.001	71.0 (61.0–82.0)	< 0.001
Death, n (%)				< 0.001		< 0.001
No	18,064 (77.4)	3,786 (62.7)	14,278 (82.6)		4,859 (80.4)	
Yes	5,267 (22.6)	2,254 (37.3)	3,013 (17.4)		1,181 (19.6)	

AST: Adjuvant Systemic Therapy; NST: Neoadjuvant Systemic Therapy; PSM: Propensity Score Matching; IQR: interquartile range.

^a^ According to Elixhauser score.

^b^ According to ICD-10.

Most patients presented no comorbidities in the first year of treatment (98.2% for NST and 97.4% for surgery). About a third of the women who underwent surgery first had tumors with invasive lesions (27.7%), whereas almost half of those who received NST first had unspecified site tumors (47.1%) (Table 1). Tumor stage at diagnosis differed between the groups, as expected. Stage III was more common among those who received NST followed by surgery (91.7%), whereas among those who underwent surgery followed by AST, this percentage was 37.2%. Additionally, 17.4% of those who underwent surgery first were diagnosed at stage I (Table 1).

Almost 80% of patients in both groups required radiotherapy (79.9% in NST *versus* 81.8% in surgery; p < 0.001). Regarding treatments received after surgery, 84.4% from the surgery first group received chemotherapy, 55.1% hormone therapy and 0.45% Anti-Her2 drugs. In the group of women received NST first, 54.9% required hormone therapy and 0.28% anti-HER2 drugs (Table 1).

Median follow-up time was 72 months (IQR = 61.0–83.0). In the matched cohorts, follow-up duration was longer for patients who underwent surgery first compared with those who received NST (71.0; IQR = 61.0–82.0 and 66; IQR = 31.0–79.0, respectively; p < 0.001). During the study period (2008–2015), the analysis showed a higher occurrence of deaths among patients who received NST first compared with those who underwent surgery first (37.3% and 19.6%, respectively; p < 0.001) (Table 1).

**Figure 2:**
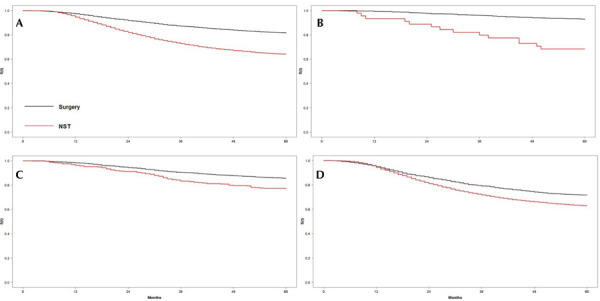
Kaplan-Meyer curves of 5-years overall survival of women with breast cancer treated by the Brazilian Unified Health System with neoadjuvant systemic treatment or surgery as first treatment from the first treatment initiation to death or censorship, overall and according to stage at diagnosis.

Note: A: full cohort; B: stage I; C: stage II; D: stage III.

OS rate of five years was 0.641 (95%CI: 0.628–0.653) for women who received NST followed by surgery and 0.816 (95%CI: 0.807–0.826) for those who underwent surgery followed by AST, with a statistically significant difference (log-rank test: p < 0.001). When stratified by stage at diagnosis, women with stage I who received NST followed by surgery had a 5-years OS of 0.684 (95%CI: 0.560–0.836) whereas those who underwent surgery followed by AST had 0.927 (95%CI: 0.911–0.942). Those with stage II presented a 5-year OS of 0.769 (95%CI: 0.730–0.809) and 0.856 (95%CI: 0.843–0.869), and those with stage III of 0.630 (95%CI: 0.617–0.643) and 0.715 (95%CI: 0.617–0.643), respectively (Figure 2).

Log-rank p < 0.001.

For both groups, older patients (70 years and older) living in northern and Midwestern Brazil, in municipalities with low and medium HDI, and self-declared Black showed lower OS probabilities. Use of hormone therapy after surgery was associated with higher survival probabilities, as well as conservative surgery instead of mastectomy (Table 2).

**Table 2:** Five-year overall survival probabilities of patients with breast cancer treated by the Brazilian Unified Health System, according to the type of the first treatment received.

	Neoadjuvant Systemic Treatment (n = 6,040)		Adjuvant Systemic Treatment (n = 6,040)	
	OS probability (95%CI)	log-rank	OS probability (95%CI)	log-rank
First treatment received	64.1% (62.8–65.3)	-	81.6% (80.7–82.6)	-
Age at treatment initiation (years)		< 0.001		< 0.001
18 to 39	61.7% (58.6–65.0)		80.9% (78.4–83.6)	
40 to 49	69.2% (67.1–71.3)		83.4% (81.8–85.1)	
50 to 59	61.8% (59.5–64.1)		81.8% (80.0–83.6)	
60 to 69	63.4% (60.6–66.4)		82.4% (80.1–84.7)	
> 70	56.5% (51.9–61.5)		71.8% (67.4–76.5)	
Region of residence		< 0.001		0.005
North	53.0% (46.7–60.3)		72.0% (65.7–79.0)	
Northeast	63.7% (61.1–66.3)		81.1% (79.1–83.3)	
Southest	64.6% (62.8–66.4)		82.3% (80.9–83.7)	
South	67.5% (64.9–70.3)		82.6% (80.4–84.8)	
Midwest	58.4% (54.1–63.1)		80.4% (76.8–84.3)	
Municipality of residence HDI		0.008		< 0.001
Low and very low	60.2% (54.5–66 .6)		73.8% (68.8–79.3)	
Medium	60.0% (57.0–63.2)		78.3% (75.8–81.0)	
High	65.0% (63.5–66.6)		82.1% (80.9–83.4)	
Very high	65.3% (62.5–68.1)		84.6% (82.6–86.6)	
Race/skin color		< 0.001		0.2
White	65.6% (64.1–67.3)		82.0% (80.8–83.3)	
Black	55.5% (51.1–60.3)		78.6% (74.6–82.8)	
Brown (Mixed-race)	63.2% (61.2–65.4)		81.5% (79.9–83.2)	
Stage at diagnosis		< 0.001		< 0.001
I	68.4% (56.0–83.6)		92.7% (91.1–94.2)	
II	76.9% (73.0–80.9)		85.6% (84.3–86.9)	
III	63.0% (61.7–64.3)		71.5% (69.7–73.5)	
Use of hormone therapy after surgery		< 0.001		< 0.001
Yes	72.5% (71.0–74.0)		89.4% (88.4–90.5)	
No	53.7% (51.8–55.6)		72.0% (70.3–73.7)	
Type of the surgery performed		< 0.001		< 0.001
Mastectomy	62.0% (60.7–63.3)		77.8% (76.4–79.3)	
Conservative	77.3% (74.5–80.3)		85.8% (84.5–87.1)	

OS: overall survival; CI: confidence interval; HDI: Human Development Index.

Shoenfeld residuals analysis of Cox model indicated that stage at diagnosis violated the proportional hazard assumptions. Thus, the model was stratified according to this variable. Higher risks were observed for women who received NST when compared to surgery first. HR was 5.13 (95%CI: 2.95–8.88) in stage I, 1.57 (95%CI: 1.27–1.95) in stage II, and 1.38 (95%CI: 1.26–1.50) in stage III (Table 3). Schoenfeld residues were not significant in the regression models assessed (p > 0.05).

**Table 3:** Hazard ratios from Cox proportional modeling according to the stage at diagnosis I, II and III.

	Stage I	Stage II	Stage III
	HR (95%CI)	HR (95%CI)	HR (95%CI)
Surgery + Adjuvant Systemic Therapy	1	1	1
Neoadjuvant Systemic Therapy + Surgery	5.13 (2.95–8.88)	1.57 (1.27–1.95)	1.38 (1.26–1.50)

HR: hazard ratio; CI: confidence interval.

Regarding the type of surgery performed, 47.88% of women who underwent surgery first had breast-conserving surgery compared with 13.36% among those who received NST first. When considering stage at diagnosis, more women with stage I underwent breast-conserving surgery compared with stages II or III in both groups (NST followed by surgery = 22.22% in stage I, 25.11% in II and 12.33% in III; surgery followed by AST = 75.78% in I, 46.84% in II and 36.06% in III).

## DISCUSSION

Women with non-metastatic breast cancer who initially underwent surgery at SUS had higher 5-year OS rates compared with those who received NST first. Patients living in northern or Midwestern Brazil, residing in municipalities with low to medium HDI, above 70 years of age and self-identifying as Black had worse 5-years OS compared with other groups. Race/skin color did not impact OS in women who underwent surgery as first treatment, which was not observed among those who received NST. Additionally, receiving hormone therapy and undergoing conservative surgery were associated with higher survival probabilities. Our findings also suggest that stage at diagnosis significantly influenced OS, with higher stages associated with lower survival rates. Notably, HR for those who underwent surgery first compared with NST was higher in patients diagnosed at stage I.

Literature consensus states that surgery plays a crucial role in cancer treatment and management. In early stages, it helps to achieve a cure, while in more advanced stages, it improves patient quality of life^8^. However, surgery encompasses a wide range of procedures^8^. At SUS, oncological surgeries are typically performed in high complexity oncology care units and centers, as well as in general hospitals accredited for high complexity oncology care^20^.

After introduction of the first Diagnostic and Therapeutic Guidelines (DDT), surgery has been the primary treatment option for non-metastatic breast cancer. For stage III, NST is recommended in some cases, particularly for tumors larger than five centimeters, with numerous or adhered axillary lymph nodes, skin or chest wall infiltration or supraclavicular lymph node involvement^21^. The updated Brazilian protocol expanded this recommendation to include stages I and II^7^. However, as DDT are not limited to technologies incorporated into SUS, treatment units have autonomy to offer the most appropriate option for each patient, which has led to the use of NST by patients in stages I and II before^7^.

Our analysis found that 0.75% of women with stage I and 7.52% with stage II received NST. A study from the United States, using a national cancer database, co-sponsored by the American Cancer Society and the Commission on Cancer of the American College of Surgeons (which covers about 70% of cancer cases in the country), found that from 2003 to 2011 4% of women treated with NST were diagnosed with stage I and 17.8% with stage II, which are higher percentages than those reported in our study^22^.

Additionally, women who underwent surgery as initial treatment had higher 5-year OS rates than those who received NST. This finding contrasts with the literature, in which studies comparing NST and surgery as first treatment have shown no difference in OS^13,14,23^. Although NST should be guided by biological factors, research indicate that socioeconomic, racial, and geospatial factors also influence its use outcomes^24^. This was somewhat reflected in our results. Women living in northern or Midwestern Brazil, residing in municipalities with low to medium HDI, had lower OS probabilities which were worse among those who received NST first. Although our study only assessed women who overcame barriers to receive treatment at SUS, these results suggest a gap in health care access in Brazil. This finding aligns with the literature which highlights the impact of socioeconomic status and disparities over time regarding access to cancer treatment^28,29^. Alves et al.^30^ found that social inequalities put individuals at a disadvantage in accessing breast cancer treatment, particularly in the North, Northeast, and Midwest regions. According to their study, areas with more vulnerable populations and higher poverty rates face significant barriers to accessing health facilities and are often more dependent on them^30^. Similarly, Ferreira et al.^31^ showed that survival is always less favorable for the most vulnerable populations, indicating inequalities in access to early diagnosis and treatment.

Although not unique to Brazil, the organization of health care facilities is often criticized^29,32^. Health facilities should ideally be distributed and allocated according to cancer incidence in different regions; but they are, by rule, defined based on population size. Silva et al.^32^ showed that the availability of cancer treatment facilities in Brazil is insufficient, both in terms of quantity and distribution. In 2017, they were located in only 173 of the country’s 5,570 municipalities, with 39.4% held by state capitals and only 12 accredited facilities in the North^32^. Additionally, patients living in rural or intermediate areas face the challenge of traveling long distances to access these services, which is a major barrier given Brazil’s vast territorial extension. This, in turn, affects both access to diagnosis and treatment, as well as continuity of care^32^. Moreover, it is important to acknowledge the disparities in the quality of services provided and the unequal access to screening and follow-up^29,32^.

Regarding the Cox regression model results, the group that underwent surgery first presented a higher risk of death, which decreased as the cancer stage increased. Although NST is often used in stage III and/or when conservative surgery or mastectomy are not viable, it is also frequently recommended for aggressive tumors such as triple-negative or HER-2-positive tumors, even in early stages, due to their responsiveness to chemotherapy and worse prognosis^33^. As expected, women at stage I who undergo surgery generally experience better survival rates compared with those at stages II or III, which may explain the higher HR observed for stage I.

About 13% of women who received NST underwent breast-conserving surgery instead of mastectomy. This finding closely aligns with existing literature, which reports a range of 18% to 35% depending on the specific context and population^10,13,23^. While NST offers the potential to downsize a tumor and make conservative surgery possible for patients initially eligible for mastectomy, most women did not undergo it. Mougalian et al.^22^, who used data from the National Cancer Database^22^, also highlighted this concern. This may be related to the severity of the cases among the women who received NST in our study^7^. In both studies, the data source used did not provide information on why mastectomy was performed more frequently in both groups^22^.

Still, when stratifying the data by stage at diagnosis, we observed that patients in stage I and II had a lower percentage of NST compared with those in stage III, for whom current guidelines strongly recommend its use^22^.

With respect to the study limitations, despite using a populational database, our results should be interpreted with caution. Only women treated at SUS, i.e., those who had access to diagnosis and treatment, were included in the analyses. Women who did not receive any treatment at SUS, as well as those who received treatment exclusively through private insurance or out-of-pocket expenses, were not included. Regarding the use of NST, women who underwent surgery first could have been treated with NST outside the SUS or as part of a series of surgeries. However, health plans only began requiring the provision of systemic treatment from 2014 onwards, so the vast majority likely did not receive it outside the SUS. Nevertheless, to mitigate the impact of selection bias in our findings, we established clear and well-defined eligibility criteria and included a control group based as closely as possible on clinical practice. Additionally, to obtain more comparable groups and reduce biases that could impact both outcome and exposure, we matched the cohorts and performed a subgroup analysis with stage at diagnosis, which is a key factor for our primary outcome. Yet, we acknowledge that other factors could have influenced the results, which we were unable to assess given our data source, as it is primarily intended for administrative purposes.

Although we used retrospective data (from 2008 to 2014) from a period during which practices have certainly evolved and new treatments have been introduced, our results provide valuable insights that can inform current health policy-making considering both the gaps and the strengths identified in this study.

## CONCLUSION

Our study suggests that women with non-metastatic breast cancer who underwent surgery first had significantly higher 5-year OS compared with those who received NST followed by surgery. Socioeconomic and demographic factors such as municipality and race/color influenced survival outcomes. Our findings emphasize the need for tailored treatment strategies and interventions to address these disparities and optimize breast cancer outcomes in Brazil.

## Data Availability

De-identified data can be made available in accordance with the Universidade Federal de Minas Gerais data-sharing policies upon reasonable request to the database holders: MLC (mcherchiglia@ufmg.br) and AAG (augustoguerrajr@ufmg.br).
